# Supplementary material for: Clinical effectiveness of drugs in hospitalized patients with COVID-19: a systematic review and meta-analysis
Source: Ther Adv Respir Dis. 2021 Mar 25;15:17534666211007214. doi: 10.1177/17534666211007214 (PMC8010807; doi:10.1177/17534666211007214)
Supplement: sj-pdf-2-tar-10.1177_17534666211007214 – Supplemental material for Clinical effectiveness of drugs in hospitalized patients with COVID-19: a systematic review and meta-analysis [file sj-pdf-2-tar-10.1177_17534666211007214.pdf]

Reviewer 1 v.1

Comments to the Author

Nice meta-analysis paper regaring regarding COVID treatment in hospitalized patients.

Some minor comments:

-Methods: page 4, line 7 "what is the clinical effectiveness of antivirals in..". Since the hypothesis that the effect of corticosteroids is more related with stopping the auto-inflammatory process that its antiviral effect, I would recommend to chage the question to "what is the clinical effectiveness of different drugs employed for COVID19 treatment in hospitalied patients" or something similar and not only focused on "antiviral"

-Results: page 7, line 1. Since here it is said "TEN aricles were finally included...", when reviweing the Figure 1, it appears ELEVEN studies ncluded in qualitative síntesis. Please, explain or fix it.

-Results: please, change IC for CI (confidence interval in english)
