# Supplementary material for: Clinical effectiveness of drugs in hospitalized patients with COVID-19: a systematic review and meta-analysis
Source: Ther Adv Respir Dis. 2021 Mar 25;15:17534666211007214. doi: 10.1177/17534666211007214 (PMC8010807; doi:10.1177/17534666211007214)
Supplement: sj-pdf-3-tar-10.1177_17534666211007214 – Supplemental material for Clinical effectiveness of drugs in hospitalized patients with COVID-19: a systematic review and meta-analysis [file sj-pdf-3-tar-10.1177_17534666211007214.pdf]

Reviewer 2 v.1

Comments to the Author

1. Correct the keywords as SARS-CoV-2 (the submitted last one).

2. Need to discuss the PICOS structure within the submitted manuscript.

3. Conclusion part must be separated from the main text.

4. Why authors included 28 days mortality? Even early, late mortality already included.

5.Citations are needed to updated, following published articles must be included within the relevant text part of the manuscript.

a. DOI: 10.4103/1995-7645.281613

b. DOI: 10.26355/eurrev_202004_20871 (PMID: 32329877)

c. DOI: 10.1016/j.arcmed.2020.05.001 (PMID: 32439198)

d.. DOI: 10.1002/jmv.25997 (PMID: 32391920)

e. DOI: 10.1002/jmv.26078 (PMID: 32462717)

f. DOI: 10.1016/j.arcmed.2020.05.009 (PMID: 32482373)

g. DOI: 10.1002/jmv.25736 (PMID: 32108359)

h. DOI: 10.1016/j.arcmed.2020.05.021 (PMID: 32532523)

i. https://doi.org/10.1016/j.imu.2020.100394

j. doi.org/10.3389/fphar.2020.01258

6. Authors stated about the PRISMA guidelines for systematic review, but not in details in the methods section, need to fix it.

7. As written ' The review protocol was registered on the PROSPERO platform ...' must be correct it and revise.

8. Recommend revising the table and figures caption to free from typological errors.
