# Supplementary material for: Clinical effectiveness of drugs in hospitalized patients with COVID-19: a systematic review and meta-analysis
Source: Ther Adv Respir Dis. 2021 Mar 25;15:17534666211007214. doi: 10.1177/17534666211007214 (PMC8010807; doi:10.1177/17534666211007214)
Supplement: sj-pdf-4-tar-10.1177_17534666211007214 – Supplemental material for Clinical effectiveness of drugs in hospitalized patients with COVID-19: a systematic review and meta-analysis [file sj-pdf-4-tar-10.1177_17534666211007214.pdf]

Supplemental file

Table 1. Search strategies

1	COVID-19
2	SARS-CoV-2
3	1 OR 2
4	hospitalized
5	admitted
6	4 OR 5
7	Remdesivir
8	Chloroquine
9	Hydroxychloroquine
10	Lopinavir
11	Ritonavir
12	Plasma
13	Dexamethasone
14	7 OR 8 OR 9 OR 10 OR 11 OR 12 OR 13
15	3 AND 6 AND 14

Table 2. Risk of bias assessment

N	Study ID	Experimental	Comparator	Outcome	Randomization process	Deviations from intended interventions	Missing outcome data	Measurement of the outcome	Selection of the reported result	Overall
1	Cao 2020	Lopinavir-Ritonavir	Standard care	Early mortality	+	?	+	–	+	–
2	Wang 2020	Remdesivir	Placebo	Early mortality	+	+	+	+	+	+
3	Cao 2020	Lopinavir-Ritonavir	Standard care	Late mortality	+	?	+	–	+	–
4	Wang 2020	Remdesivir	Placebo	Late mortality	+	+	+	+	+	+
5	Cao 2020	Lopinavir-Ritonavir	Standard care	28 days mortality	+	?	+	–	+	–
6	Hung 2020	Lopinavir-Ritonavir-Rivabirin-Interferon	Lopinavir-Ritonavir	30 days mortality	?	?	+	+	+	!
7	Wang 2020	Remdesivir	Placebo	28 days mortality	+	+	+	+	+	+
8	Borba 2020	Chloroquine high dosage	Chloroquine low dosage	28 days mortality	+	+	+	+	+	+

+ Low risk

? Some concerns

– High risk

9	Horby 2020	Dexamethasone	Standard care	28 days mortality	+	?	+	+	+	!
10	Li 2020	Convalescent plasma	Standard care	28 days mortality	–	–	+	+	+	–
11	Tomazini 2020	Dexamethasone	Standard care	28 days mortality	–	?	+	+	+	–
12	Cao 2020	Lopinavir-Ritonavir	Standard care	Clinical improvement 7 days	+	?	+	–	+	–
13	Hung 2020	Lopinavir-Ritonavir-Rivabirin-Interferon	Lopinavir-Ritonavir	Clinical improvement 7 days	?	?	+	+	+	!
14	Wang 2020	Remdesivir	Placebo	Clinical improvement 7 days	+	+	+	+	+	+
15	Li 2020	Convalescent plasma	Standard care	Clinical improvement 7 days	–	–	+	+	+	–
16	Spinner 2020	Remdesivir	Standard care	Clinical improvement 7 days	–	?	+	+	+	–
17	Cao 2020	Lopinavir-Ritonavir	Standard care	Clinical improvement 14 days	+	?	+	–	+	–
18	Wang 2020	Remdesivir	Placebo	Clinical improvement 14 days	+	+	+	+	+	+
19	Li 2020	Convalescent plasma	Standard care	Clinical improvement 14 days	–	–	+	+	+	–
20	Spinner 2020	Remdesivir	Standard care	Clinical improvement 14 days	–	?	+	+	+	–
21	Cao 2020	Lopinavir-Ritonavir	Standard care	Clinical improvement 28 days	+	?	+	–	+	–
22	Wang 2020	Remdesivir	Placebo	Clinical improvement 28 days	+	+	+	+	+	+
23	Li 2020	Convalescent plasma	Standard care	Clinical improvement 28 days	–	–	+	+	+	–
24	Spinner 2020	Remdesivir	Standard care	Clinical improvement 28 days	–	?	+	+	+	–
25	Cao 2020	Lopinavir-Ritonavir	Standard care	Duration of ventilation	+	?	+	–	+	–

26	Wang 2020	Remdesivir	Placebo	Duration of ventilation	+	+	+	+	+	+
27	Cao 2020	Lopinavir-Ritonavir	Standard care	Duration of oxygen support	+	?	+	–	+	–
28	Wang 2020	Remdesivir	Placebo	Duration of oxygen support	+	+	+	+	+	+
29	Cao 2020	Lopinavir-Ritonavir	Standard care	Hospital stay	+	?	+	–	+	–
30	Hung 2020	Lopinavir-Ritonavir-Rivabirin-Interferon	Lopinavir-Ritonavir	Hospital stay	?	?	+	+	+	!
31	Wang 2020	Remdesivir	Placebo	Hospital stay	+	+	+	+	+	+
32	Cao 2020	Lopinavir-Ritonavir	Standard care	Virus clearance	+	?	–	–	+	–
33	Hung 2020	Lopinavir-Ritonavir-Rivabirin-Interferon	Lopinavir-Ritonavir	Virus clearance	?	?	–	+	+	–
34	Wang 2020	Remdesivir	Placebo	Virus clearance	+	+	–	+	+	–
35	Chen 2020	Hydroxychloroquine	Standard care	Virus clearance	?	?	+	+	+	!
36	Gautret 2020	Hydroxychloroquine	Standard care	Virus clearance	–	–	+	+	+	–
37	Tang 2020	Hydroxychloroquine	Standard care	Virus clearance	–	?	+	+	+	–
38	Li 2020	Convalescent plasma	Standard care	Virus clearance	–	–	+	+	+	–
39	Cao 2020	Lopinavir-Ritonavir	Standard care	Severe adverse events	+	?	+	+	+	!
40	Hung 2020	Lopinavir-Ritonavir-Rivabirin-Interferon	Lopinavir-Ritonavir	Severe adverse events	?	?	+	+	+	!
41	Wang 2020	Remdesivir	Placebo	Severe adverse events	+	+	+	+	+	+
42	Chen 2020	Hydroxychloroquine	Standard care	Severe adverse events	?	?	+	+	+	!

43	Tang 2020	Hydroxychloroquine	Standard care	Severe adverse events	−	?	+	+	+	−
44	Borba 2020	Chloroquine high dosage	Chloroquine low dosage	Severe adverse events	+	+	+	+	+	+
45	Spinner 2020	Remdesivir	Standard care	Severe adverse events	−	?	+	+	+	−
46	Tomazini 2020	Dexamethasone	Standard care	Severe adverse events	−	?	+	+	+	−

Meta-analysis

Figure S.1. Forest plot of drugs employed in hospitalized patients with SARS-CoV-2 infection.

Comparison: Remdesivir versus Standard care or Placebo.

Outcome: Severe adverse events.

	Remdesivir [Experimental]		Standard care or placebo [Control]			Odds Ratio
**Study or Subgroup**	**Events**	**Total**	**Events**	**Total**	**Weight**	**IV, Fixed, 95% CI**
Spinner 2020	19	394	18	200	48.8%	0.51 [0.26, 1.00]
Wang 2020	28	155	20	78	51.2%	0.64 [0.33, 1.23]
**Total (95% CI)**		**549**		**278**	**100.0%**	**0.57 [0.36, 0.92]**
Total events	47		38			
Heterogeneity: Chi² = 0.22, df = 1 (P = 0.64); I² = 0%						
Test for overall effect: Z = 2.33 (P = 0.02)						

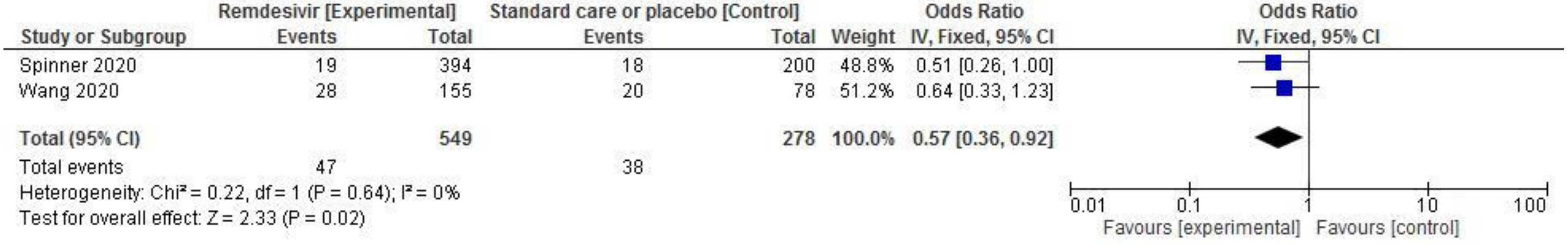

Figure S.2. Forest plot of drugs employed in hospitalized patients with SARS-CoV-2 infection.

Comparison: Hydroxychloroquine versus Standard care.

Outcome: Virological clearance at day 7.

	Hydroxychloroquine [Experimental]		Standard care [Control]			Odds Ratio
**Study or Subgroup**	**Events**	**Total**	**Events**	**Total**	**Weight**	**IV, Random, 95% CI**
Chen 2020	33	75	43	75	39.9%	0.58 [0.31, 1.12]
Gautret 2020	14	20	2	16	32.9%	16.33 [2.80, 95.26]
Tang 2020	13	15	14	15	27.2%	0.46 [0.04, 5.75]
**Total (95% CI)**		**110**		**106**	**100.0%**	**1.64 [0.17, 15.67]**
Total events	60		59			
Heterogeneity: Tau² = 3.21; Chi² = 12.30, df = 2 (P = 0.002); I² = 84%						
Test for overall effect: Z = 0.43 (P = 0.67)						

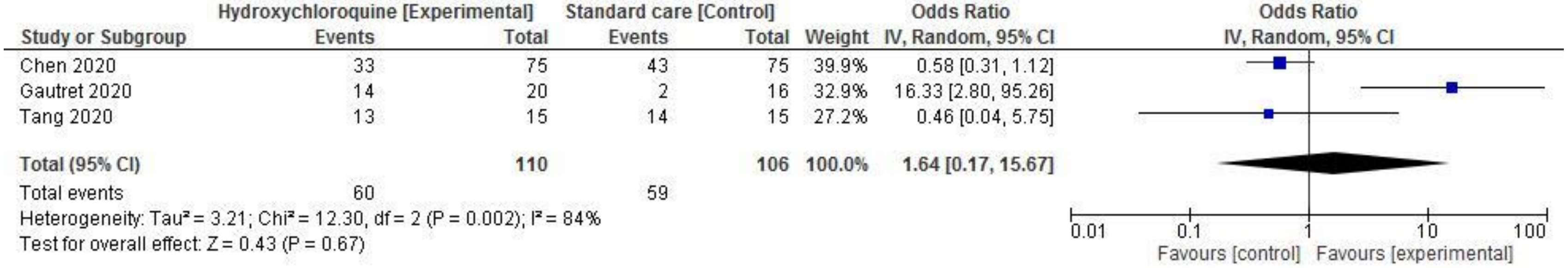

Figure S.3. Forest plot of drugs employed in hospitalized patients with SARS-CoV-2 infection.

Comparison: Hydroxychloroquine versus Standard care.

Outcome: Severe adverse events.

	HydroxychloroExperimental		Control			Odds Ratio
**Study or Subgroup**	**Events**	**Total**	**Events**	**Total**	**Weight**	**IV, Fixed, 95% CI**
Chen 2020	4	15	3	15	76.2%	1.45 [0.26, 8.01]
Tang 2020	2	75	0	75	23.8%	5.14 [0.24, 108.81]
**Total (95% CI)**		**90**		**90**	**100.0%**	**1.96 [0.44, 8.71]**
Total events	6		3			
Heterogeneity: Chi² = 0.50, df = 1 (P = 0.48); I² = 0%						
Test for overall effect: Z = 0.89 (P = 0.37)						

Odds Ratio
IV, Fixed, 95% CI

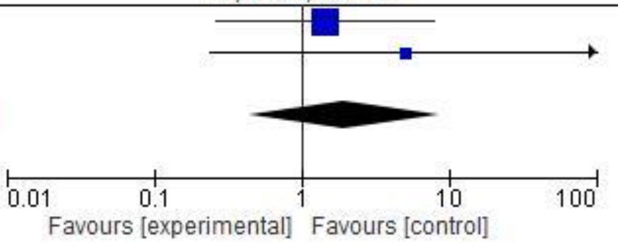

Table 3. Excluded studies with reasons

Author	Reference	Reason for excluding
Lecronier 2020	[1]	retrospective
Saiz Rodriguez 2020	[2]	protocol
Duska 2020	[3]	protocol
Doggrell 2020	[4]	expert opinion article
Bosch Barrera 2020	[5]	monograph
Liu 2020	[6]	protocol
Singh 2020	[7]	monograph
Maillart 2020	[8]	case report
Jean 2020	[9]	monograph
Abena 2020	[10]	monograph
Ahmad 2020	[11]	monograph
Plaze 2020	[12]	monograph
Wang 2020	[13]	protocol
Lother 2020	[14]	protocol
Thibodeaux 2020	[15]	case report
Shen 2020	[16]	case report
Martínez 2020	[17]	monograph
Kakodkar 2020	[18]	review
Ye 2020	[19]	retrospective
Lai 2020	[20]	monograph
Gao 2020	[21]	letter
Şimşek Yavuz 2020	[22]	review
Monteiro 2020	[23]	letter
Yousefifard 2020	[24]	review
Viveiros Rosa 2020	[25]	review
González 2020	[26]	protocol
Sivapalam 2020	[27]	protocol

References of excluded articles

1. Lecronier M, Beurton A, Burrel S, et al. Comparison of hydroxychloroquine, lopinavir/ritonavir, and standard of care in critically ill patients with SARS-CoV-2 pneumonia: an opportunistic retrospective analysis. *Crit Care* 2020; 24: 418.

2. Saiz-Rodríguez M, Peña T, Lázaro L, et al. Outpatient treatment of COVID-

19 with steroids in the phase of mild pneumonia without the need for admission as an opportunity to modify the course of the disease: A structured summary of a randomised controlled trial. *Trials* 2020; 21: 632.

3. Duška F, Waldauf P, Halačová M, et al. Azithromycin added to hydroxychloroquine for patients admitted to intensive care due to coronavirus disease 2019 (COVID-19)—protocol of randomised controlled trial AZIQUINE-ICU. *Trials* 2020; 21: 631.

4. Doggrell SA. Does lopinavir measure up in the treatment of COVID-19? *Expert Opin Investig Drugs* 2020; 1–4.

5. Bosch-Barrera J, Martin-Castillo B, Buxó M, et al. Silibinin and SARS-CoV-2: Dual Targeting of Host Cytokine Storm and Virus Replication Machinery for Clinical Management of COVID-19 Patients. *J Clin Med* 2020; 9: 1770.

6. Liu X, Chen H, Shang Y, et al. Efficacy of chloroquine versus lopinavir/ritonavir in mild/general COVID-19 infection: a prospective, open-label, multicenter, randomized controlled clinical study. *Trials* 2020; 21: 622.

7. Singh AK, Singh A, Shaikh A, et al. Chloroquine and hydroxychloroquine in the treatment of COVID-19 with or without diabetes: A systematic search and a narrative review with a special reference to India and other developing countries. *Diabetes Metab Syndr Clin Res Rev* 2020; 14: 241–246.

8. Maillart E, Leemans S, Van Noten H, et al. A case report of serious haemolysis in a glucose-6-phosphate dehydrogenase-deficient COVID-19 patient receiving hydroxychloroquine. *Infect Dis (Auckl)* 2020; 52: 659–661.

9. Jean S-S, Hsueh P-R. Old and re-purposed drugs for the treatment of COVID-19. *Expert Rev Anti Infect Ther* 2020; 1–5.

10. Abena PM, Decloedt EH, Bottieau E, et al. Chloroquine and Hydroxychloroquine for the Prevention or Treatment of COVID-19 in Africa: Caution for Inappropriate Off-label Use in Healthcare Settings. *Am J Trop Med Hyg* 2020; 102: 1184–1188.

11. Ahmad M., Dasgupta A, Chopra S. Remdesivir. RNA-dependent RNA polymerase inhibitor, Treatment of Ebola virus infection, Treatment of SARS-CoV-2 infection (COVID-19). *Drugs Future* 2020; 45: 369.

12. Plaze M, Attali D, Petit A-C, et al. Repositionnement de la chlorpromazine dans le traitement du COVID-19 : étude reCoVery. *Encephale* 2020; 46: S35–S39.

13. Wang Y, Zhou F, Zhang D, et al. Evaluation of the efficacy and safety of intravenous remdesivir in adult patients with severe COVID-19: study protocol for a phase 3 randomized, double-blind, placebo-controlled, multicentre trial. *Trials* 2020; 21: 422.

14. Lother SA, Abassi M, Agostinis A, et al. Post-exposure prophylaxis or pre-emptive therapy for severe acute respiratory syndrome coronavirus 2 (SARS-CoV-2): study protocol for a pragmatic randomized-controlled trial. *Can J Anesth Can d'anesthésie* 2020; 67: 1201–1211.

15. Thibodeaux K, Speyrer M, Raza A, et al. Hyperbaric oxygen therapy in preventing mechanical ventilation in COVID-19 patients: a retrospective case series. *J Wound Care* 2020; 29: S4–S8.

16. Shen C, Wang Z, Zhao F, et al. Treatment of 5 Critically Ill Patients With COVID-19 With Convalescent Plasma. *JAMA* 2020; 323: 1582.

17. Martinez MA. Compounds with Therapeutic Potential against Novel Respiratory 2019 Coronavirus. *Antimicrob Agents Chemother*; 64. Epub ahead of print 9 March 2020. DOI: 10.1128/AAC.00399-20.

18. Kakodkar P, Kaka N, Baig M. A Comprehensive Literature Review on the Clinical Presentation, and Management of the Pandemic Coronavirus Disease 2019 (COVID-19). *Cureus*. Epub ahead of print 6 April 2020. DOI: 10.7759/cureus.7560.

19. Ye XT, Luo YL, Xia SC, et al. Clinical efficacy of lopinavir/ritonavir in the treatment of Coronavirus disease 2019. *Eur Rev Med Pharmacol Sci* 2020; 24: 3390–3396.

20. Lai C-C, Shih T-P, Ko W-C, et al. Severe acute respiratory syndrome coronavirus 2 (SARS-CoV-2) and coronavirus disease-2019 (COVID-19): The epidemic and the challenges. *Int J Antimicrob Agents* 2020; 55: 105924.

21. Gao J, Tian Z, Yang X. Breakthrough: Chloroquine phosphate has shown apparent efficacy in treatment of COVID-19 associated pneumonia in clinical studies. *Biosci Trends* 2020; 14: 72–73.

22. ŞİMŞEK YAVUZ S, ÜNAL S. Antiviral treatment of COVID-19. *TURKISH J Med Sci* 2020; 50: 611–619.

23. Monteiro WM, Brito-Sousa JD, Baía-da-Silva D, et al. Driving forces for COVID-19 clinical trials using chloroquine: the need to choose the right research questions and outcomes. *Rev Soc Bras Med Trop*; 53. Epub ahead of print 2020. DOI: 10.1590/0037-8682-0155-2020.

24. Yousefifard M, Zali A, Mohamed Ali K, et al. Antiviral therapy in management of COVID-19: a systematic review on current evidence. *Arch Acad Emerg Med* 2020; 8: e45.

25. Rosa SGV, Santos WC. Clinical trials on drug repositioning for COVID-19 treatment. *Rev Panam Salud Pública* 2020; 44: 1.

26. González R, García-Otero L, Pons-Duran C, et al. Hydroxychloroquine efficacy and safety in preventing SARS-CoV-2 infection and COVID-19

disease severity during pregnancy (COVID-Preg): a structured summary of a study protocol for a randomised placebo controlled trial. *Trials* 2020; 21: 607.

27. Sivapalan P, Ulrik CS, Bojesen RD, et al. Proactive Prophylaxis With Azithromycin and HydroxyChloroquine in Hospitalised Patients With COVID-19 (ProPAC-COVID): A structured summary of a study protocol for a randomised controlled trial. *Trials* 2020; 21: 513.
