# Supplementary material for: Clinical effectiveness of drugs in hospitalized patients with COVID-19: a systematic review and meta-analysis
Source: Ther Adv Respir Dis. 2021 Mar 25;15:17534666211007214. doi: 10.1177/17534666211007214 (PMC8010807; doi:10.1177/17534666211007214)
Supplement: sj-pdf-1-tar-10.1177_17534666211007214 – Supplemental material for Clinical effectiveness of drugs in hospitalized patients with COVID-19: a systematic review and meta-analysis [file sj-pdf-1-tar-10.1177_17534666211007214.pdf]

Author Response

Reviewer: 1

Comments to the Author

Nice meta-analysis paper regaring regarding COVID treatment in hospitalized patients.
Some minor comments:

-Methods: page 4, line 7 "what is the clinical effectiveness of antivirals in..". Since the hypothesis that the effect of corticosteroids is more related with stopping the auto-inflammatory process that its antiviral effect, I would recommend to chage the question to "what is the clinical effectiveness of different drugs employed for COVID19 treatment in hospitalied patients" or something similar and not only focused on "antiviral"

R: The review question was corrected
-Results: page 7, line 1. Since here it is said "TEN aricles were finally included...", when reviweing the Figure 1, it appears ELEVEN studies ncluded in qualitative síntesis. Please, explain or fix it.
R: Yes, it was our mistake, it was fixed. Eleven studies were finally included
-Results: please, change IC for CI (confidence interval in english)
R: It was changed.

Reviewer: 2

Comments to the Author
1. Correct the keywords as SARS-CoV-2 (the submitted last one).
R: It was corrected in all sections of the manuscript.
2. Need to discuss the PICOS structure within the submitted manuscript.
R: It was included in the results and in the discussion section.

3. Conclusion part must be separated from the main text.
R: We have divided and placed the Conclusion as a new section after the Discussion.
4. Why authors included 28 days mortality? Even early, late mortality already included.
R: Yes, it is right. We had to include the 28 days mortality due to the heterogeneity of the reporting in the Clinical trials. In the limitations (Discussion), we have included the following paragraph:
Another limitation is that not all studies assessed mortality outcomes in the same way. Some only evaluated early mortality, others evaluated late mortality, and others, mortality at day 28; this is another crucial point on heterogeneity.

5.Citations are needed to updated, following published articles must be included within the relevant text part of the manuscript.
a. DOI: 10.4103/1995-7645.281613
b. DOI: 10.26355/eurrev_202004_20871 (PMID: 32329877)
c. DOI: 10.1016/j.arcmed.2020.05.001 (PMID: 32439198)
d.. DOI: 10.1002/jmv.25997 (PMID: 32391920)
e. DOI: 10.1002/jmv.26078 (PMID: 32462717)

f. DOI: 10.1016/j.arcmed.2020.05.009 (PMID: 32482373)
g. DOI: 10.1002/jmv.25736 (PMID: 32108359)
h. DOI: 10.1016/j.arcmed.2020.05.021 (PMID: 32532523)
i. https://doi.org/10.1016/j.imu.2020.100394
j. doi.org/10.3389/fphar.2020.01258

R: Recommended citations were included in the following paragraph:
"While there is no vaccine available, in the meantime other studies are still being developed all over the world from different therapeutic focus, as part of a joint effort by all academics, clinicians, and scientists around the world 32–41."

6. Authors stated about the PRISMA guidelines for systematic review, but not in details in the methods section, need to fix it.
R: Prisma guidelines are not a methodology itself, but rather a set of 27 items in a checklist that serves as a minimal set of items to help reporting systematic reviews and meta-analysis.
In other words, these are elements that must be presented in a systematic review, a meta-analysis, or both.
For this reason, it is not recommended to detail the PRISMA guidelines as methods, it is only a matter of following a checklist to consider that a systematic review is complete and that none of the elements cited in the list of 27 items is missing.

7. As written ' The review protocol was registered on the PROSPERO platform ...' must be correct it and revise.
R: We have corrected the information about the changes derived form the registered protocol, in the PROSPERO Platform as follows:
Since the dynamics of the COVID-19 pandemic changed over the weeks, it was necessary to make an adjustment in the initial protocol, which only contemplated the elderly.
The adjustment consisted of expanding the patients in the PICOS structure to include all hospitalized adult patients, not just older adults.
The final results and publications derived from this protocol included the following PICOS structure:
• Patients: Adults hospitalized with a diagnosis of SARS-CoV-2 infection.
• Intervention: Treatment with the following drugs: remdesivir, chloroquine, hydroxychloroquine, lopinavir, ritonavir, dexamethasone, and convalescent plasma.
• Comparison: Standard care or placebo.
• Outcomes: Early mortality, late mortality, 28 days mortality, clinical improvement at seven days, clinical improvement at 14 days, clinical improvement at 28 days, duration of ventilation (days), duration of oxygen support (days), duration of hospitalization (days), virological clearance, and severe adverse events.
• Studies (type of): Clinical trials published in peer-reviewed journals.

This correction will be available online in the Platform as soon as it is approved by the PROSPERO reviewers.

8. Recommend revising the table and figures caption to free from typological errors.
R: All archives were scanned, and spelling/typing errors were fixed.
